# 1357. Patterns of Extragenital Gonorrhea and Chlamydia Testing at a Community-Based Academic Emergency Department in Columbus, Ohio

**DOI:** 10.1093/ofid/ofab466.1549

**Published:** 2021-12-04

**Authors:** Francisco M Magaña, Mohammad Mahdee Sobhanie, Carlos Malvestutto, Jose A Bazan, Ashley Lipps

**Affiliations:** The Ohio State University Wexner Medical Center, Columbus, Ohio

## Abstract

**Background:**

Sexually transmitted infections (STI), including gonorrhea (GC) and chlamydia (CT), are on the rise in the U.S. and emergency department (ED) visits for STI-related complaints are common. The ED plays a key role in testing for GC/CT. In addition to testing genital sites for GC/CT, the Centers for Disease Control and Prevention (CDC) recommends extragenital testing (oral/rectal) based on sexual history and exposure. In this study, we reviewed the proportion of extragenital GC/CT tests performed at a community-based academic ED in Columbus, Ohio.

**Methods:**

This study was a retrospective chart review of all GC/CT tests performed at the Ohio State University Hospitals East ED from November 1, 2018 to November 1, 2020. Clinical and demographic information was collected for all patients who received extragenital GC/CT testing, including symptoms, test results, and documentation of sexual practices. A random convenience sample of 100 patients who only had genital GC/CT testing performed was also reviewed.

**Results:**

Of the 5644 GC/CT tests performed during the study period, only 364 (6.4%) were from extragenital sites, which included 311 (5.5%) from oral and 53 (< 1%) from rectal sites. Of the 100 patients reviewed who did not have extragenital GC/CT testing performed, only 5 (5%) had documentation of sexual practices, compared with 177/311 (56.9%) of those who had oral testing and 27/31 (50.94%) who had rectal testing performed. In the cohort of 100 patients who did not receive extra genital testing 28% were male and average age was 29. In the group who received extragenital testing 40% were male and average age was 30. The most common complaint across all groups was genital discharge

**Conclusion:**

Despite the substantial number of CG/CT tests performed in the ED, only a very small proportion were from extragenital sites. Interventions are needed to identify and overcome barriers to extragenital GC/CT testing in the ED.

**Disclosures:**

**Mohammad Mahdee Sobhanie, M.D.**, **Regeneron** (Scientific Research Study Investigator)**Regeneron** (Scientific Research Study Investigator, Was a sub-investigator for Regeneron 2066 and 2069) **Carlos Malvestutto, M.D.**, **Lilly** (Scientific Research Study Investigator)**Regeneron Inc.** (Scientific Research Study Investigator)**ViiV Healthcare** (Advisor or Review Panel member)

